# Preferred, small-scale foraging areas of two Southern Ocean fur seal species are not determined by habitat characteristics

**DOI:** 10.1186/s12898-019-0252-x

**Published:** 2019-09-11

**Authors:** Mia Wege, P. J. Nico de Bruyn, Mark A. Hindell, Mary-Anne Lea, Marthán N. Bester

**Affiliations:** 1 Mammal Research Institute, Department of Zoology & Entomology, University of Pretoria, Private Bag X20, Hatfield, Pretoria, 0028 South Africa; 2 Institute for Marine and Antarctic Studies, University of Tasmania, 20 Castray Esplanade, Battery Point, Hobart, TAS 7004 Australia; 3 Antarctic Climate and Ecosystems Cooperative Research Centre, University of Tasmania, Hobart, TAS 7004 Australia

**Keywords:** *Arctocephalus*, Boosted regression tree, Foraging behaviour, Foraging segregation, Machine learning, Marion Island, Niche, Sympatry

## Abstract

**Background:**

To understand and predict the distribution of foragers, it is crucial to identify the factors that affect individual movement decisions at different scales. Individuals are expected to adjust their foraging movements to the hierarchical spatial distribution of resources. At a small local scale, spatial segregation in foraging habitat happens among individuals of closely situated colonies. If foraging segregation is due to differences in distribution of resources, we would expect segregated foraging areas to have divergent habitat characteristics.

**Results:**

We investigated how environmental characteristics of preferred foraging areas differ between two closely situated Subantarctic fur seal (*Arctocephalus tropicalis*) colonies and a single Antarctic fur seal (*A. gazella*) colony that forage in different pelagic areas even though they are located well within each other’s foraging range. We further investigated the influence of the seasonal cycle on those environmental factors. This study used tracking data from 121 adult female Subantarctic and Antarctic fur seals, collected during summer and winter (2009–2015), from three different colonies. Boosted Regression Tree species distribution models were used to determine key environmental variables associated with areas of fur seal restricted search behaviour. There were no differences in the relative influence of key environmental variables between colonies and seasons. The variables with the most influence for each colony and season were latitude, longitude and magnitude of sea-currents. The influence of latitude and longitude is a by-product of the species’ distinct foraging areas, despite the close proximity (< 25 km) of the colonies. The predicted potential foraging areas for each colony changed from summer to winter, reflecting the seasonal cycle of the Southern Ocean. The model predicted that the potential foraging areas of females from the three colonies should overlap, and the fact they do not in reality indicates that factors other than environmental are influencing the location of each colony’s foraging area.

**Conclusions:**

The results indicated that small scale spatial segregation of foraging habitats is not driven by bottom-up processes. It is therefore important to also consider other potential drivers, e.g. competition, information transfer, and memory, to understand animal foraging decisions and movements.

## Background

The presence and abundance of prey play a crucial role in the distribution of marine predators living in highly seasonal environments. However, marine ecosystems are dynamic and complex [1] with resources constantly moving in three-dimensional space. Scale-dependent physical and biological processes determine the distribution of nutrients and subsequent productivity [2], abundance of grazers, prey, and consequently, predators [3–5]. Bathymetry, sea-surface temperature, frontal regions, meso-scale eddies, wind and currents are examples of environmental variables that influence marine predators [6–10]. Marine predators, high profile components of marine ecosystems, feed at a range of trophic levels within a variety of marine habitats, and are sensitive to shifts in the aforementioned bottom-up processes [11]. Alterations to the productivity, abundance, and distribution of lower trophic level organisms therefore affect many aspects of top-predator life history and ultimately influence their population growth [12, 13]. This leads to the common assumption that facets of their behaviour, health, reproductive output, and subsequent population growth status are indicative of the productivity and quality of food within the ocean [13, 14]. As a result, marine predators are thought to be ideal sentinels to monitor changes in marine ecosystems [13].

Despite the major influence of external, environmental bottom-up processes on foraging behaviour of predators, these are not the only determinants of predator foraging behaviour. Other drivers, such as age (experience), sex, species interactions, and breeding status that are a few aspects of demography that contribute to variations in foraging behaviour and subsequent population dynamics [15–21]. However, the dictators of some foraging behaviours are still unknown or currently only theoretical. Spatial segregation between conspecifics from neighbouring colonies is one such example. Initially, spatial segregation between individuals from distant colonies were thought to occur because of differential bottom-up processes that drive foraging preferences [22, 23]. For spatially restricted species, such as central-place foragers, the distance required to travel from the colony to the foraging areas of distant colonies would be energetically too expensive and therefore force individuals to forage closer to their own colonies [24, 25]. However, recent research indicate that even closely situated colonies that are well within each other’s foraging range also segregate spatially. Such segregation occurs in, for example, Adélie penguins (*Pygoscelis adeliae*) [26, 27], Northern fur seals (*Callorhinus ursinus*) [28, 29], Macaroni penguins (*Eudyptes chrysolophus*) [30], and Magellanic penguins (*Spheniscus magellanicus*) [31]. Current hypotheses suggest that this small-scale spatial segregation is driven by competitive exclusion, which is then further enhanced by private information (i.e. memory) and public information, where the colony acts as an information centre and individuals inadvertently transfer knowledge to conspecifics either at the colony when they are observed departing to or returning from a direction (i.e. the information centre hypothesis [32]. However, to date it has not been tested how common environmental bottom-up drivers of these closely situated colonies that forage in disparate regions, differ from each other.

Sympatric female Subantarctic fur seals (*Arctocephalus tropicalis;* SAFS) and Antarctic fur seals (*A. gazella;* AFS) at sub-Antarctic Marion Island have species- and colony-specific foraging areas. They also change these foraging areas from summer to winter [33], maintain minimal overlap with foragers from neighbouring colonies, despite the colonies being situated well within the travelling range of both species. We aim to understand how environmental characteristics of preferred foraging areas differ between two SAFS colonies and an AFS colony, at Marion Island; and how the seasonal cycle of the Southern Ocean modulates these characteristics. We quantify the at-sea distribution and foraging habitats of female SAFS and AFS from three closely situated colonies across summer and winter. We hypothesize that the environmental variables associated with each of the three colonies’ foraging locations will not differ and that model predicted potential foraging areas among the three colonies overlap. However, due to the commanding role the seasonal cycle in the Southern Ocean plays on primary productivity, and prey distribution and abundance, we expect environmental variables associated with foraging locations for each colony to change from summer to winter. We ask three key questions: (1) does the preferred foraging areas of female fur seals from the three study colonies differ in environmental indicators? (2) Do these environmental indicators change from summer to winter? (3) Are the environmental indicators of preferred foraging areas present in areas associated with foraging of the neighbouring colonies?

## Results

At-sea locations for 121 lactating females are presented from 44 AFS from a high-density colony (summer: 24, winter: 20; hereafter HD_AFS); 40 SAFS females from the high-density colony (summer: 19, winter: 21; hereafter HD_SAFS) and 37 SAFS females from the low-density colony (summer: 15, winter: 22; hereafter LD_SAFS) between 2009 and 2015. The data comprise 617 foraging trips (range: 1–16 trips per female) of which 560 (91%) were complete and 36 (9%) incomplete (Additional file 1: Fig. S1). There were 93,500 location estimates after state-space model filtering. Of these, 39,066 (41.78%) location estimates were classified as restricted search areas and, 54,367 (58.18%) as transit: only 67 (< 1%) locations could not be classified and were subsequently removed from further analyses.

### Determining coverage of true colony foraging areas

Summer individuals from all three colonies reached an asymptote of the number of new grid cells added within < 10 individuals whereas winter individuals never fully reached an asymptote (Additional file 1: Fig. S2). Winter females’ inflexion point of the number of new 0.25° × 0.25° cells added was between 20 and 25 grid cells, with HD_AFS showing the least decrease in number of cells added with each new tracked female added (Additional file 1: Fig. S2).

### Species distribution models

The best learning rate, tree complexity, and bagging fraction that resulted in the least amount of residual deviance were learning rate = 0.0005, tree complexity = 5, and bagging fraction = 0.5, respectively (Table 1).

**Table 1:** Final parameters used for each of the boosted regression tree models

Colony_season	Number of trees	Training set size	Variables excluded	AUC	Deviance
HD_AFS winter	500,000	5000	Frontal region	0.96	0.50
HD_AFS summer	329,440	5000	Frontal region	0.97	0.32
HD_SAFS winter	499,400	5000	Frontal region	0.96	0.54
HD_SAFS summer	160,000	2000	Frontal region	0.93	0.67
LD_SAFS winter	500,000	5000	Frontal region	0.97	0.44
LD_SAFS summer	205,060	2000	Frontal region	0.93	0.68

*HD_SAFS* high-density Subantarctic fur seal colony, *LD_SAFS* the low-density Subantarctic fur seal colony, *HD_AFS* the high-density Antarctic fur seal colony, *AUC* the area under curve of the receiver operating characteristic

**Fig. 1:**
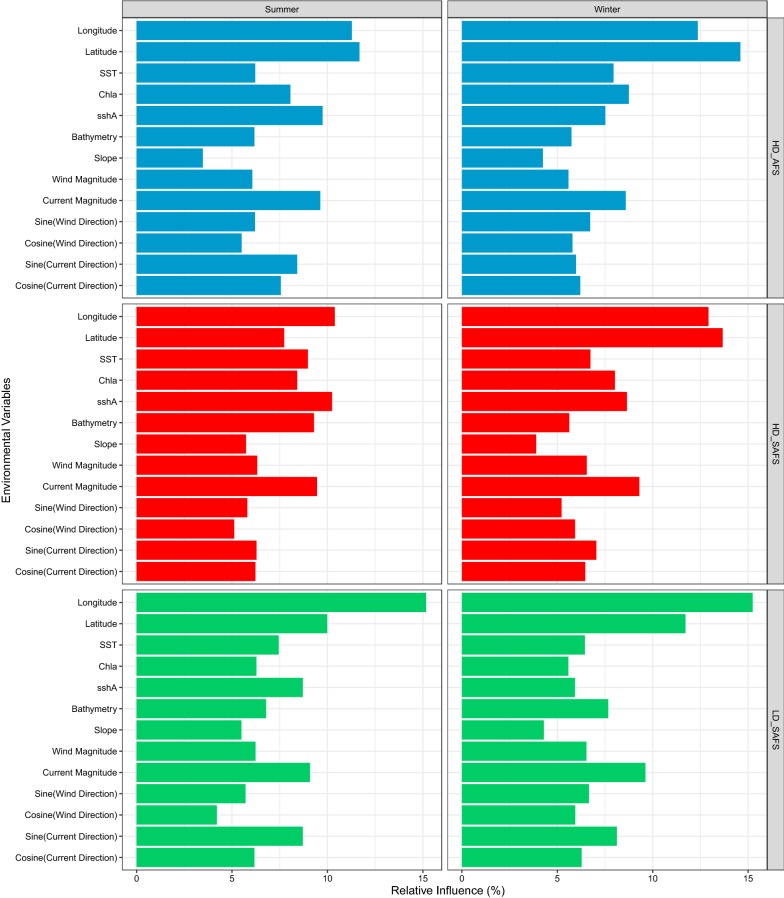
The relative influence (%) of environmental variables of the final Boosted Regression Tree models. *HD_SAFS* high-density Subantarctic fur seal colony, *LD_SAFS* the low-density Subantarctic fur seal colony, and *HD_AFS* the high-density Antarctic fur seal colony

All six final BRT models include all but one (mean seasonal frontal region) of the co-variates. The relative influence of each of the variables differed very little among the models (Fig. 1). Longitude and latitude were among the top three ranked environmental variables for all models, except the HD_SAFS summer model (Fig. 1). Aside from latitude and longitude, ocean current magnitude was the only variable which was within the top five variables of all BRT models. The other five top environmental variables, all contributing the most to relative influence of the final BRT models, were SST, bathymetry, Chla, and sshA and the sine of ocean current direction (i.e. the ‘eastness’ of the current).

**Fig. 2:**
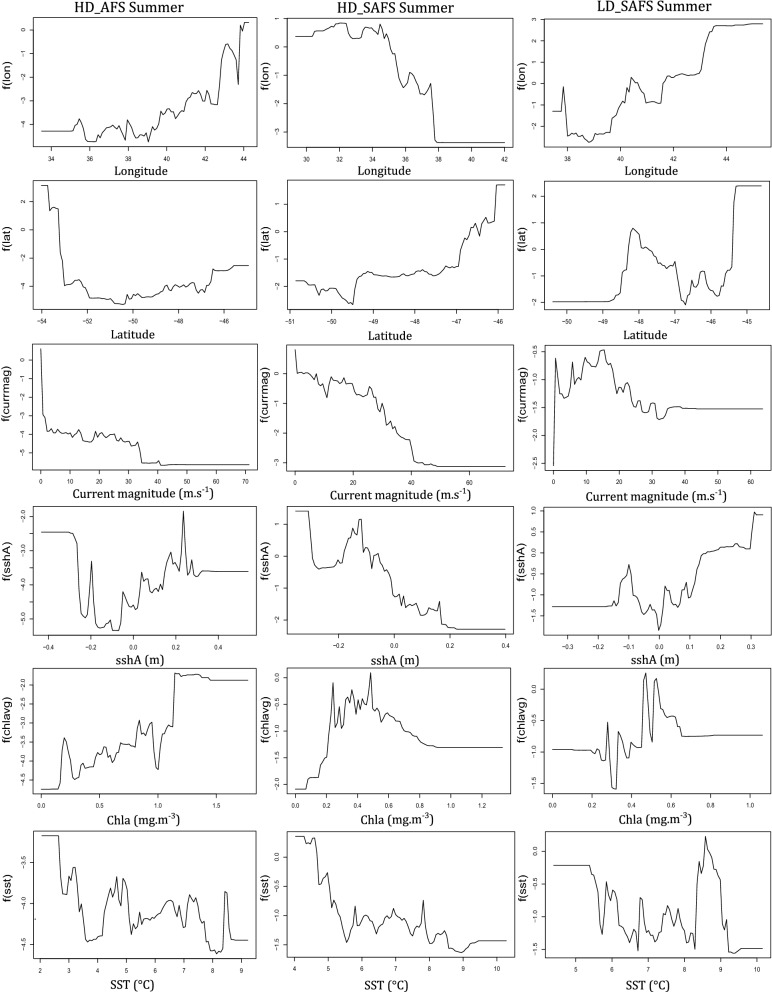
Response curves of summer females from each of the three colonies of only the top environmental variables for the high-density Subantarctic fur seal colony (HD_SAFS), the low-density Subantarctic fur seal colony (LD_SAFS), and the high-density Antarctic fur seal colony (HD_AFS)

**Fig. 3:**
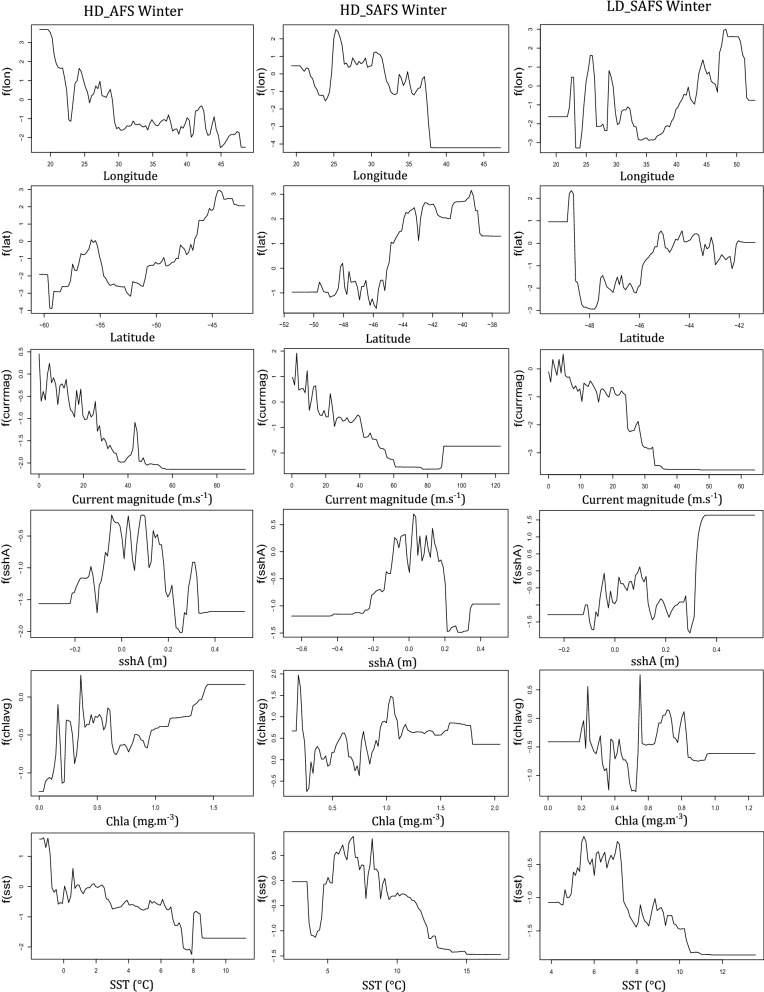
Response curves of winter females from each of the three colonies of only the top environmental variables for the high-density Subantarctic fur seal colony (HD_SAFS), the low-density Subantarctic fur seal colony (LD_SAFS), and the high-density Antarctic fur seal colony (HD_AFS)

Although the relative contribution of the environmental predictor variables did not differ greatly between colonies and seasons, the response curves (relationships) between the probability of restricted search and the predictor variables differed among colonies and seasons. The response curves for most of the behavioural activity predictors for the seals were non-linear (Figs. 2, 3; Additional file 1: Figs. S3–S8). The relationship between the probability of restricted search and the predictor variables differed between colonies with the biggest difference in the response curves of latitude and longitude between colonies. The relationship between restricted search areas and current magnitude was negative across all seasons and colonies (Figs. 2, 3). Between seasons the biggest differences were the values of the predictor variables where the peaks and troughs of restricted search probability occurred (Figs. 2, 3; Additional file 1: Figs. S3–S8).

### Predicted potential area of restricted search regions

**Fig. 4:**
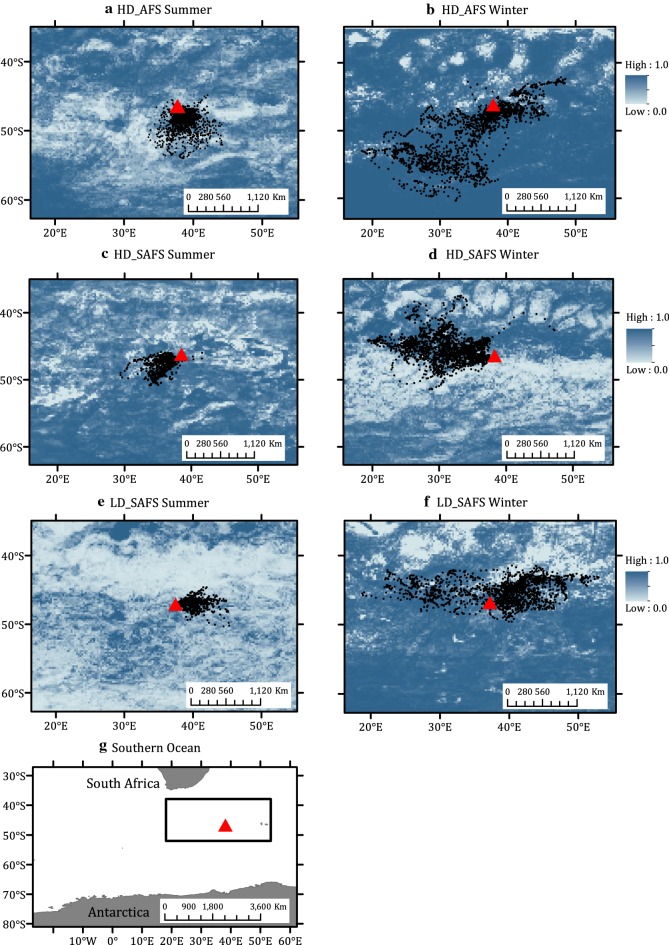
Predicted foraging habitat suitability (preferred areas for restricted search) in the region surrounding Marion Island (grey triangle) for: **a**, **b** the high-density Antarctic fur seal colony; (HD_AFS); **c**, **d** the high-density Subantarctic fur seal colony (HD_SAFS); **e**, **f** and the low-density Subantarctic fur seal colony (LD_SAFS), during summer and winter respectively. State-space model estimated restricted search locations are indicated by the black dots. **g** The location within the Southern Ocean of the area represented in figures **a**–**f** are given by the rectangle

During summer months, potential restricted search regions were predicted to be available within the areas utilized by seals from each colony as well as the other two colonies’ foraging domains (Fig. 4a, c, e). HD_AFS and LD_SAFS females mostly only have potential available restricted search regions to the south of Marion Island (Fig. 4a, e), whereas HD_SAFS had options available to areas off the north-west of the island too (Fig. 4c). Some HD_SAFS females spent time in a non-predicted region due-west of Marion Island (Fig. 4c). During winter, HD_AFS females had regions available all-around Marion Island except for some patches north and north-west of the island (Fig. 4b). HD_SAFS females had potential restricted search areas available to the west, north-west, and east of Marion Island (Fig. 4d), whereas LD_SAFS females had areas all around Marion Island available, except for a region north-east of the island (Fig. 4f).

## Discussion

Predators are sensitive to bottom-up processes, however, here, we demonstrated that there are no pronounced differences in environmental variables associated with distinct, neighbouring foraging areas of lactating SAFS and AFS. Furthermore, the relative contribution of these variables associated with restricted search changed little from summer to winter. Model predicted potential foraging areas did change from summer to winter, indicating that seasonal fluctuations and spatial movements of prey aggregations and its availability are likely driving seasonal changes in fur seal movements. The models predicted that the potential foraging areas of females from the three colonies should overlap, and that these did not, indicates that factors other than environmental characteristics influence the location of female foraging areas for each colony.

Females from all colonies and seasons displayed less restricted search in areas with stronger ocean current. Faster current speed precludes the formation of closed-circulation cells and the subsequent retention of water bodies leading to upwelling and enhanced primary production [34]. Marion Island lies in a region of enhanced eddy kinetic energy caused by the eastward flowing Antarctic Circumpolar Current’s collision with the South-west Indian Ridge upstream of the island [35–37]. During summer, females from the HD_AFS and LD_SAFS colonies had higher probability of restricted search in areas with positive sshA, whereas HD_SAFS had more restricted search in negative sshA (Fig. 2). During winter, females from all three colonies foraged more in regions of positive sshA. Positive sshA are indicative of cold-core cyclonic eddies that spin up to the surface. Cyclonic eddies have higher concentrations of chla around the edges of the eddies [36] and during winter could provide pockets of productivity within an otherwise resource depleted habitat. In this area of the Southern Ocean, juvenile southern elephant seals (*Mirounga leonina*) [38] were also shown to avoid “intense eddy features” and similarly in the summer foraged in areas with positive sshA values. In the winter however, juvenile elephant seals foraged at the edges of eddies, where sshA slope values are high [38]. Similarly, grey-headed albatross (*Thalassarche chrysostoma*) also foraged in sshA; during chick-rearing period in particular around the edges of these anomalies formed in the South-west Indian Ridge [39].

Although sea-surface temperature contributed little to relative influence on all six models (Figs. 2, 3) there are clear peaks and troughs in the response curves at 4 °C and 8 °C. These are the surface isotherms where the Polar Front and the sub-Antarctic Front are typically found [35]. Seasonal mean frontal region was the only variable dropped from the BRT models, which suggested little or no contribution to restricted search behaviour. However, diving behaviour of both fur seal species at Marion Island are influenced by frontal regions [40]. Foraging behaviour of SAFS females from neighbouring Prince Edward Island (46°37′47″S; 37°56′17″E) and distant Amsterdam Island (37°49′33″S; 77°33′17″E) are also influenced by frontal regions, specifically the sub-Antarctic Front [41–43]. It is therefore possible that averaging frontal regions across multiple years (this study) did not capture the scale at which frontal regions influence SAFS and AFS foraging behaviour at Marion Island.

Wind strength and direction contributed very little to all models. This observation contrasts recent findings of winter tracking of females from a low-density AFS (LD_AFS) colony, also on Marion Island [44]. Arthur et al. [45] used time-spent in an area to match the error uncertainty associated with geo-location sensing tags. It is unlikely that the dissimilarities between this study and [44] is due to differences between species, seasons or colonies given that this study found little differences among the three other colonies at the same island location. Furthermore, winter foraging areas between the HD_AFS colony and the LD_AFS colony overlapped greatly [18, 44, 55]. Given that environmental correlates influence predator foraging behaviour differently across a hierarchy of spatial scales [46], this difference might be due to larger scale areas used by [44] to infer restricted search areas.

Ultimately, the differences in relative influence of key environmental variables between colonies or seasons were small and nuanced. Latitude and longitude, the two most important variables in the final model, were also the two variables of which the response curves differed the most among colonies. Although predictions using interpolations of habitat models are useful to expand on the potential habitats of marine species for conservation practices [11, 25, 44], it should also be taken into account that these are not always “realistic” foraging areas for species spatio-temporally restricted in their movements away from their colonies. In this study, habitat predicted available foraging areas overlapped among colonies and seasons. We suggest that the aforementioned, and the dominating influence of latitude and longitude, is potentially a by-product of the intrinsically-driven colony-preferred foraging directions of the fur seal females [55]. At a small local scale (i.e., at the same island), commonly measured environmental variables, as a proxy for some bottom-up processes, are not the drivers of spatial segregation of core habitat utilisation areas between colonies; otherwise seals would have foraged in a neighbouring colony’s core foraging areas due to the negligible swimming distances between them. Environmental correlates certainly do influence predator foraging behaviour at a larger regional scale and when comparing environmental drivers of predator foraging behaviour between distantly located islands it would mostly differ due to the local environment experienced by the predators at each of the separate island colonies [24, 47, 48]. This only gives one an indication of the dominant environmental drivers of prey-aggregations within the regional scale surrounding that colony. For example, during winter the preferred travelling directions of king penguins (*Aptenodytes patagonicus*) from the Falkland Islands are dominated by the local, northward-flowing Falkland Current [49]; or the influence of the shelf-break of the Kerguelen Plateau on the diving behaviour of Antarctic fur seals [10]. Pinaud and Weimerskirch [37] showed how seven different Indian Ocean Procellariformes species (albatrosses and petrels) changed searching behaviour either between species or in response to different habitat types (intra-species). This was a broad-scale comparison between three distant islands (Amsterdam, Crozet and Kerguelen) and concluded that we need to study movements at smaller scales in relation to resource distribution to understand scale-dependent foraging distribution of predators. Here, at a small local scale—the core foraging areas of fur seal females from three colonies that forage in separate areas, but still experience the same local conditions (present study), are not only driven by prey aggregations [50]. Several studies have found that individuals from neighbouring colonies of central-place foragers segregate from each other despite being situated well within each other foraging range [28, 29, 51–53]. Often the density or size of the colony has an influence on the home range size of a colony, i.e. offspring from smaller colonies are lighter presumably because parents had to make longer foraging trips further afield [26, 51]. This is explained through intra-specific competition, where the larger colony outcompetes the smaller colony [19, 51]. However, density-dependent competition does not always drive spatial segregation between neighbouring colonies, including here for fur seals at Marion Island [[55],^52^]. Current hypotheses suggest that information transfer, memory, and learned behaviours could drive this spatial segregation [28, 32, 51, 52, 54], although the actual mechanisms behind this remain largely unstudied [28].

The model predicted potential foraging areas shifted from summer to winter for all study colonies on Marion Island (this study), akin to SAFS from Prince Edward Island, where predicted foraging regions also changed seasonally to areas further afield from the study colony in winter [41]. As environmental conditions change with the seasons (e.g. water temperatures decreasing, shifting wind patterns and current changes), this would most likely cause the locations of prey aggregations to shift from summer to winter [56, 57] and subsequently result in the shift of potential foraging habitat.

Predicted potential foraging areas of HD_AFS females span almost the entire region surrounding Marion Island. This result should be interpreted with caution given that the cumulative information analysis suggested that the available tracking data for HD_AFS winter females did not adequately capture the spatial use patterns of the entire colony. Therefore, we have little confidence in the HD_AFS winter predicted foraging areas and more tracking data are needed to accurately predict alternative foraging areas around Marion Island.

## Conclusions

The results indicated that there are no pronounced differences in environmental variables associated with distinct, neighbouring foraging areas of lactating SAFS and AFS. Furthermore, the relative contribution of these variables associated with restricted search changed little from summer to winter. Model predicted potential foraging areas did change from summer to winter, implicating the seasonal fluctuations and spatial movements of prey aggregations and its availability. The models predicted that the potential foraging areas of females from the three colonies should overlap, and that these did not, indicates that small scale spatial segregation of foraging habitats is not driven by bottom-up processes. It is therefore important to also consider other potential drivers, e.g. competition, information transfer, and memory, to understand animal foraging decisions and movements.

## Methods

**Fig. 5:**
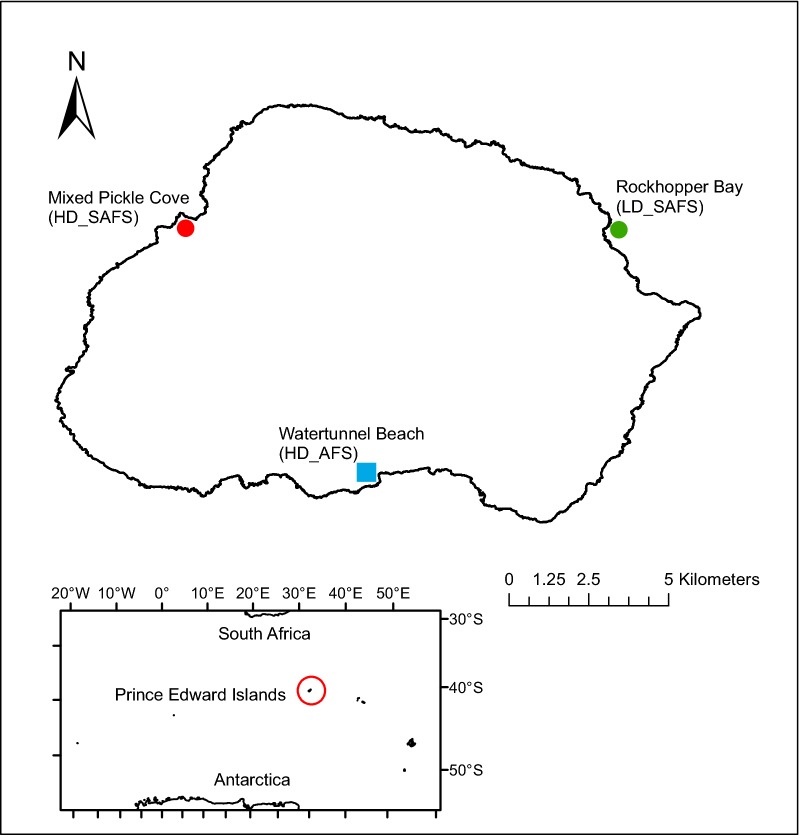
The location of the three study colonies on Marion Island. *HD_SAFS* high-density Subantarctic fur seal colony, *LD_SAFS* low-density Subantarctic fur seal colony, *HD_AFS* high-density Antarctic fur seal colony. The Prince Edward Islands’ location in the Southern Ocean is shown in the insert

The three breeding colonies are situated around the coastline of sub-Antarctic Marion Island. Watertunnel Beach, the high-density AFS colony, is situated on the south coast (46°58′6.4″S; 37°44′39.73″E, hereafter HD_AFS); Mixed Pickle Cove, the high-density SAFS colony, is situated on the west coast (46°52′15.88″S; 37°38′18.27″E, hereafter HD_SAFS) and Rockhopper Bay, the low-density SAFS colony is situated on the northeast coast of Marion Island (46°52′13.33″S; 37°51′25.34″E, hereafter LD_SAFS; Fig. 5). Fur seal densities at colonies are relative to Marion Island’s fur seal population size and were determined using the distance along the coastline of the beach (HD_AFS = 34 m; HD_SAFS = 40 m; LD_SAFS = 300 m) and pup production at the beaches (HD_AFS = ~ 1100 pups; HD_SAFS = ~ 500 pups; LD_SAFS = ~ 100 pups; [58, 59]. The HD_SAFS colony is 21.35 km from the LD_SAFS colony and 25.10 km from the HD_AFS colony; the LD_SAFS and HD_AFS colonies are situated 23.38 km from each other.

### Instrumentation

Breeding adult females seen suckling were selected at random, caught in a hoop-net, and physically restrained. A female either received a Sirtrack Argos-linked platform transmitter terminal (Kiwisat 101; to measure at-sea location) together with a Wildlife Computers MK9 time-depth recorder (Redmond, Washington, USA); or alternatively, a female only received an Argos-linked (CLS, Toulouse, France) Wildlife Computers MK10 SPLASH tag. Additional file 1: Table S1 summarizes the spatio-temporal deployment protocol. Devices were attached to the dorsal midline pelage just below the scapulae of the animal using a double component, quick-setting epoxy resin (Araldite AW2101, CIBA-GEIGY Ltd.). All summer deployments were made around the median pupping date for both species (AFS = 6 December; SAFS = 18 December; [45], while winter deployments were made post-moult between late April to early May. Females were recaptured after ~ 4 months and the devices removed. Summer deployments spanned mid-December to early March and winter deployments late April to early August (these dates varied based on battery life, or whether a female or her pup survived). Winter deployments on AFS were done just prior to a female’s pup weaning and before adult females disperse for the post-lactation foraging trip (AFS lactation period 110 days [December–April]) [60]. Conversely, SAFS females have a lactation period of 300 [December–October] days and were still nursing a pup throughout the winter tracking period [60].

### Filtering tracking data by means of state-space models

To account for the inherent observation error relayed through the global Argos satellite system we fitted a two-state, behavioural switching, state-space model to the tracks [61, 62]. State-space models filtered out flawed location estimates and provided location estimates at a 2.5 h interval. State-space models also assigns a behavioural mode of restricted search, likely to be foraging locations, or transit. Prior to analyses, all seal tracks were split into individual foraging trips. Females were considered ‘on-land’ when the dive recorder measured a dry-period, or for those individuals without diving records, through visual inspection of location estimates by determining whether a point was on land or at sea. Bayesian state-space models were fitted for each foraging trip using Markov chain Monte Carlo in ‘*rjags’* [63], via the ‘*bsam’* package [61, 62] implemented in programme R [64]. A hierarchical formulation allows for estimation of parameters for multiple animals and their individual foraging trips [61]. A time step of 2.5 h was used based on the median number of Argos location points per day (9–10 points per day). Two Markov chains were run in parallel, each of 50,000 iterations, using only every 200th value, while the first 10,000 values (i.e., burn-in) were excluded. Diagnostic plots were used to assess converging and appropriate mixing of the two Markov chains [65].

### Determining coverage of true colony foraging areas

To determine representativeness of tracking data per colony and season we estimated curves of the cumulative number of grid cells visited for each new individual tracked. The order of females was randomized over 100 iterations and averaged across the number of individuals added. This was done at a 0.25° × 0.25° resolution [44, 66]. This provides an assessment of the minimum number of animals needed to represent the spatial distribution patterns of females from each colony and season adequately. Given that females from the three study colonies segregate [55] and that foraging areas between summer and winter differ [33], this process was done separately for each season within each colony. The average number of individuals was plotted against a spline and the asymptote is indicative of the number of individuals required to broadly characterize the movements of the female population (Additional file 1: Fig. S2).

### Environmental predictor variables

Environmental variables used to characterise foraging areas were sea-surface height anomalies (sshA; [m]), sea-surface temperature (sst; [°C]), chlorophyll-a concentration (chla; [mg m^−3^]), bathymetry [m], the slope of the ocean floor [°], wind direction [°] and strength [m s^−1^], oceanic current direction [°] and strength [m s^−1^], and mean seasonal frontal region. Current flow direction and wind direction are both circular variables and to interpret these in a linear fashion, we decomposed both variables into ‘northness’ and ‘eastness’ using cosine and sine transformations of the mean direction in radians, respectively [67]. All of these environmental characteristics affect marine top-predator foraging behaviour [6–10, 18]. Variables were extracted from the Australian Antarctic Data Centre using the R package ‘*raadtools’* [68]. Additional file 1: Table S2 provides the data source as well as spatial and temporal resolution of all environmental variables.

## Oceanic frontal regions

Each location point was assigned to one of 8 inter-frontal zones, similar to [40]. We used weekly frontal positions between 1992 and 2009 [69, 70] and calculated the average inter-frontal zone for each cell, within a given month of the year across a 0.5° × 0.5° grid. This serves as a long-term average position of fronts in the Southern Ocean in a monthly timeframe [40]. The frontal zones are: (i) south of Antarctic Circumpolar Current Front—South, (ii) Antarctic Circumpolar Current to Polar Front—South, (iii) Polar Front, (iv) Polar Front to sub-Antarctic Front, (v) sub-Antarctic front, (vi) sub-Antarctic Front—North to sub-Antarctic Zone, (vii) sub-Antarctic Zone to sub-Tropical Zone—South and (viii) north of sub-Tropical Zone-South.

### Species distribution models

We used boosted regression tree models (BRT) to examine the influence of the environmental variables, as well as the latitude and longitude of each location, on the behavioural state of each location. As central-place foragers, fur seals are limited by the distance they can travel away from their colony. Including latitude and longitude as covariates in the model takes the spatial movement limitation of the species into account and constrains the model accordingly. A BRT is a machine learning technique that combines regression trees and a boosting algorithm [71, 72]. Models were constructed using the ‘*gbm’* package [73] in R [64] using additional code of [74]. Given the binomial distribution of the response variable (restricted search *vs.* travelling), we made use of a Bernoulli error structure for the loss function. A BRT requires the following parameters to be fit: (1) the learning rate or shrinkage, which determines the contribution of each tree to the growing model, (2) tree complexity that controls the number of interactions in the BRT, (3) a subsampling rate (bagging fraction), which is the proportion of the training data set used to select variables, (4) cross-validation, which specifies the number of times to randomly divide the data for model fitting and validation (we chose a tenfold cross-validation process), and (5) the number of iterations or number of trees required to minimize the predictive deviance [71]. The following five parameters were adjusted recursively to maximise model performance, with initial parameter values, based on initial parameters suggested by [74], are given in brackets: (1) the number of observations to use as the model training and evaluation dataset (training dataset = 1000 observations), (2) the number of trees to be fit (10,000), (3) the bag fraction (0.5), (4) the learning rate (0.05), and (5) tree complexity (5). Once the best value for each parameter was determined, unimportant variables were sequentially dropped, similar to backward step-wise variable selection, using model simplification methods from [74]. The area under the receiver operating characteristic curve was used as the performance measure [75]. It measures how well the model correctly distinguishes between restricted search and travelling location points, with values closest to one considered the best model. We created separate models for each of the seasons (summer vs. winter) for each of the three colonies (HD_AFS, HD_SAFS, and LD_SAFS). This was done instead of including colony and season as terms in the model because it would require a high tree complexity (interaction terms between colony, season and each of the environmental predictors) and would complicate convergence of the final model.

### Predicting suitable foraging habitat

The final BRT models were used to predict potential restricted search areas within the broader region around Marion Island. The goal was to determine whether each colony’s model predicted potential restricted search regions overlaps with observed foraging locations of the neighbouring colonies. To make predictions of appropriate restricted search regions, each of the relevant environmental variables within the final BRT models were averaged across all study years, for the 4 months’ duration of each season of tracking data analyses (i.e. summer: December–March 2010–2015; winter: May–August 2009–2014). Given that not all environmental variables were available in the same spatial resolution (Additional file 1: Table S2), all final environmental raster were resampled to a 0.25° × 0.25° grid resolution using the ‘*raster*’ package in R [64, 76]. The potential effects of preferred latitudinal and longitudinal travelling areas as well as distance from the colony from the prediction models were excluded to focus model predicted potential restricted search areas based only on environmental variables. To do this, two background grid files (i.e. rasters) were created for longitude and latitude respectively, with only 1′s (representing presence), at a 0.25° × 0.25° grid resolution, for the prediction models. We used the *‘predict.gbm’* function in the R library’*gbm*’ [73] to identify suitable restricted search regions following instructions and code provided by [74].

## Supplementary information

**Additional file 1.** Supplementary figures and tables: Preferred, small-scale foraging areas of two Southern Ocean fur seal species are not determined by habitat characteristics.

## Data Availability

Tracking data used in this study is available at: 10.6084/m9.figshare.9657494.v1.

## Abbreviations

SAFS: Subantarctic fur seal (*Arctocephalus tropicalis*)
AFS: Antarctic fur seal (*Arctocephalus gazella*)
HD_SAFS: high-density Subantarctic fur seal colony
LD_SAFS: low-density Subantarctic fur seal colony
HD_AFS: high-density Antarctic fur seal colony
sshA: sea-surface height anomaly
sst: sea-surface temperature
Chla: chlorophyll a concentration
BRT: boosted regression tree model
