# Supplementary material for: Preferred, small-scale foraging areas of two Southern Ocean fur seal species are not determined by habitat characteristics
Source: BMC Ecol. 2019 Sep 11;19:36. doi: 10.1186/s12898-019-0252-x (PMC6739983; doi:10.1186/s12898-019-0252-x)
Supplement: Supplementary file 1 — Additional file 1. Supplementary figures and tables: Preferred, small-scale foraging areas of two Southern Ocean fur seal species are not determined by habitat characteristics. [file 12898_2019_252_MOESM1_ESM.docx]

**Preferred, small-scale foraging areas of two Southern Ocean fur seal species are not determined by habitat characteristics**

Additional Table S1: Spatio-temporal deployment of satellite telemetry devices deployed. HD_SAFS = High=density Subantarctic fur seal colony (*Arctocephalus tropicalis*), LD_SAFS = the low-density Subantarctic fur seal colony, and HD_AFS = the high-density Antarctic fur seal colony (*A. gazella*).

|  | **HD_AFS** | | **HD_SAFS** | | **LD_SAFS** | |
| --- | --- | --- | --- | --- | --- | --- |
|  | Summer | Winter | Summer | Winter | Summer | Winter |
| **2009** | 0 | 0 | 0 | 4 | 0 | 5 |
| **2010** | 4 | 5 | 0 | 5 | 0 | 6 |
| **2011** | 5 | 5 | 5 | 5 | 5 | 3 |
| **2012** | 5 | 4 | 7 | 4 | 4 | 3 |
| **2013** | 4 | 3 | 5 | 3 | 4 | 2 |
| **2014** | 3 | 3 | 2 | 0 | 1 | 3 |
| **2015** | 3 | 0 | 0 | 0 | 3 | 0 |


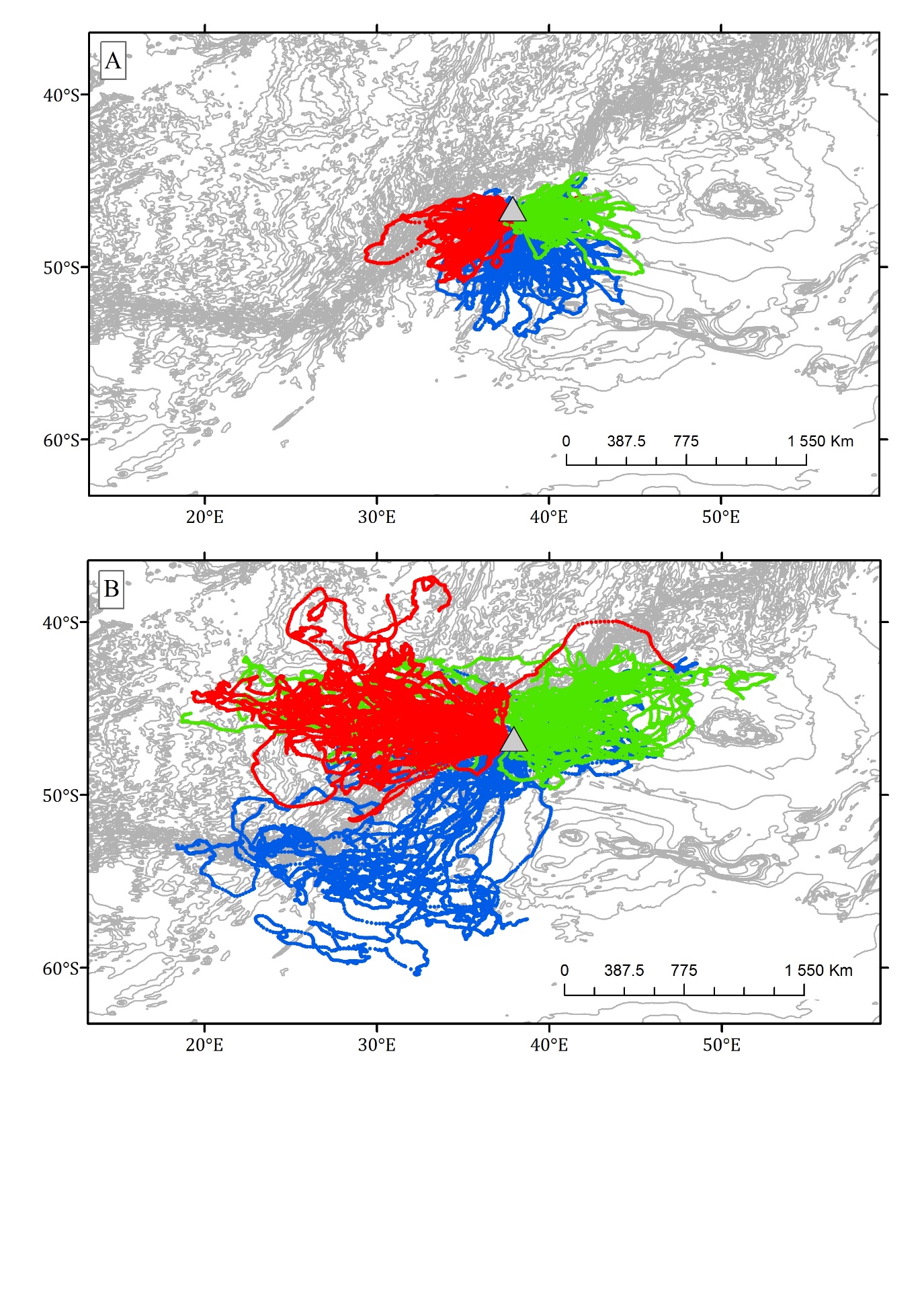


Additional Figure S1: Mean estimated at-sea locations of 121 female Subantarctic and Antarctic fur seals from Marion Island, 2009-2015 during a) summer and b) winter. Green dots represent the low-density Subantarctic fur seal colony, red dots the high-density Subantarctic fur seal colony, and blue dots the high-density Antarctic fur seal colony. Marion Island is shown in figures A and B as a grey triangle. At-sea data are presented with a 1 arc-min bathymetry overlay.

Additional Table S2: Product type (data source), temporal and spatial resolution of the various environmental variables used in Boosted Regression Tree models.

| **Environmental variable** | **Product type** | **Temporal Resolution** | **Spatial Resolution** |
| --- | --- | --- | --- |
| chlA - chlorophyll *a* concentration (mgm^-3^) | MODIS^a^ | 3 month average | 0.5° x 0.5° |
| SST - sea-surface temperature (°C) | NOAA Optimum Interpolation daily Sea Surface Temperature^b^ | weekly average | 0.25° x 0.25° |
| Bathymetry (m) | gebco_08 | NA | 0.083° x 0.083° |
| Ocean floor slope | Calculated from GEBCO-08 database | NA | 0.083° x 0.083° |
| sshA - sea-surface height anomaly | AVISO^c^ | Daily | 0.25° x 0.25° |
| Oceanic fronts | Sokolov & Rintoul (2009a,b)^d^ | Seasonal 6-month average, 1992-2009 | 0.5° x 0.5° |
| Wind (magnitude & direction) | NOAA National Centre for Environmental Prediction Reanalysis Information (NCEP2)^e^ | Daily | 0.25° x 0.25° |
| Current (magnitude & direction) | AVISO^f^ | Daily | 0.25° x 0.25° |

^a^ MODIS: <http://oceancolor.gsfc.nasa.gov/>

^b^ OI-daily: http://www.ncdc.noaa.gov/oa/climate/research/sst/oi-daily.php

^c^ AVISO: http://www.aviso.altimetry.fr/en/data/products/sea-surface-height-products.html

^d^ Sokolov, S. & Rintoul, S.R. (2009a) Circumpolar structure and distribution of the Antarctic Circumpolar Current fronts: 1. Mean circumpolar paths. Journal of Geophysical Research, 114, C11018.
Sokolov, S. & Rintoul, S.R. (2009b) Circumpolar structure and distribution of the Antarctic Circumpolar Current fronts: 2. Variability and relationship to sea surface height. Journal of Geophysical Research, 114, C11019.

^e^ NOAA http://www.esrl.noaa.gov/psd/data/gridded/data.ncep.reanalysis.html

^f^AVISO: http://www.aviso.altimetry.fr/en/data/products/windwave-products.html

^g^ All data were reprojected into 0.25 X 0.25° pixels

*
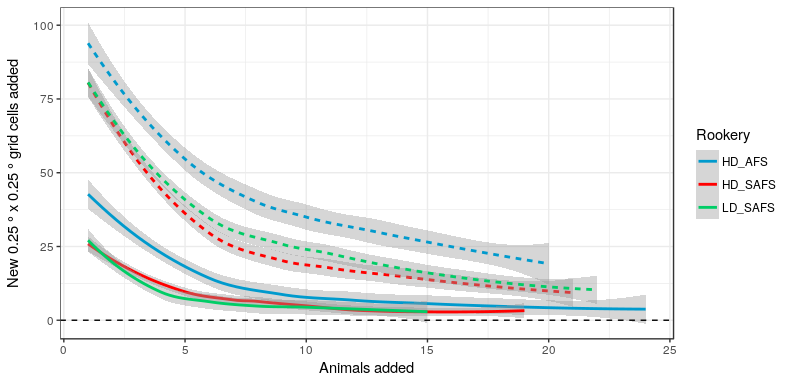
*

Additional *Figure S2: The average (± SE) number of additional 0.25°x0.25 ° grid cells visited with each new animal tracked during summer (solid lines) and winter (dashed lines) for the high-density Subantarctic fur seal colony (HD_SAFS), low-density Subantarctic fur seal colony (LD_SAFS), and high-density Antarctic fur seal colony (HD_AFS).*


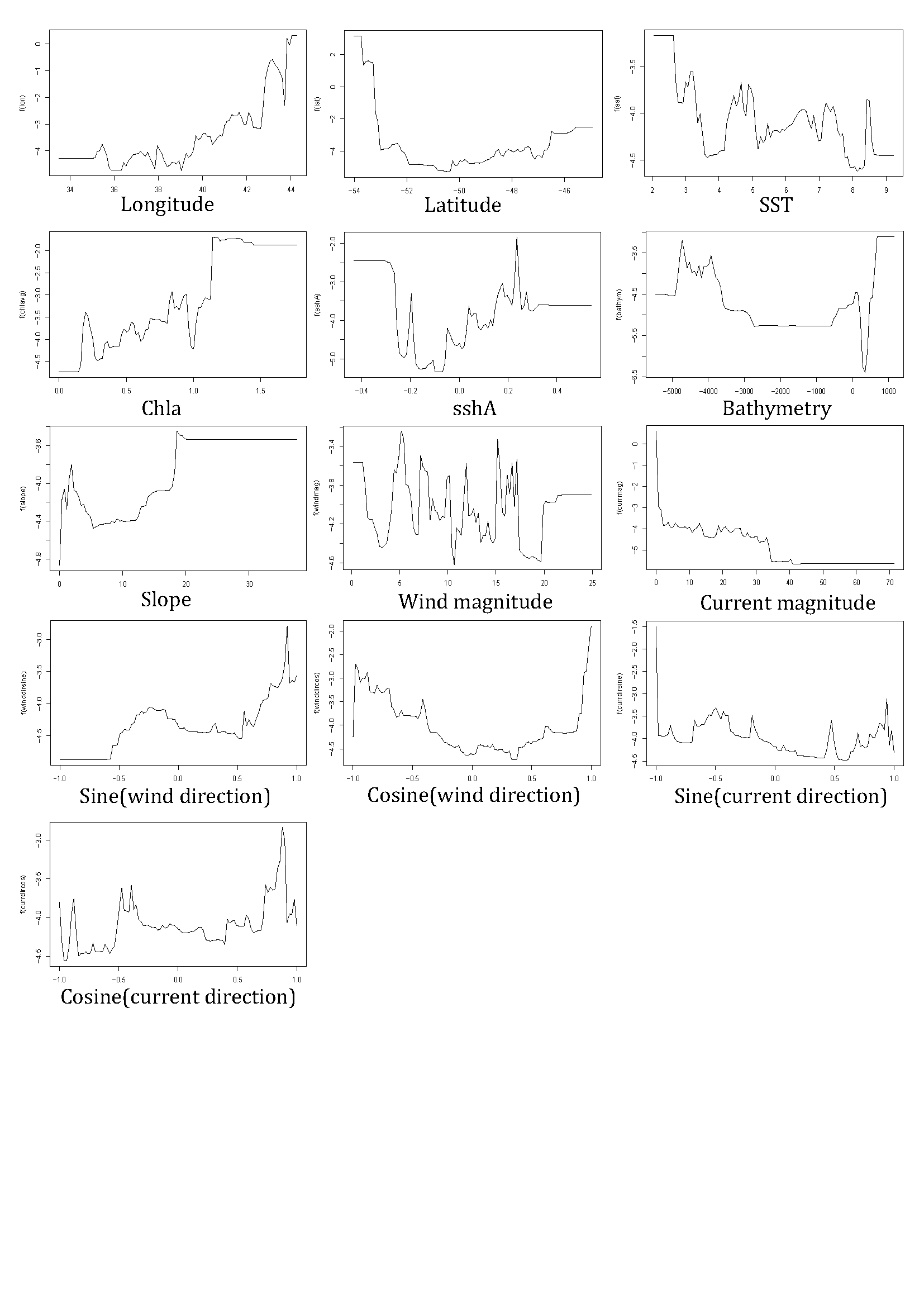


Additional Figure S3: Summer high-density Antarctic fur seal (*Arctocephalus gazella*; HD_AFS) females’ response curves of each of the environmental predictor variables for the final Boosted Regression Tree model.


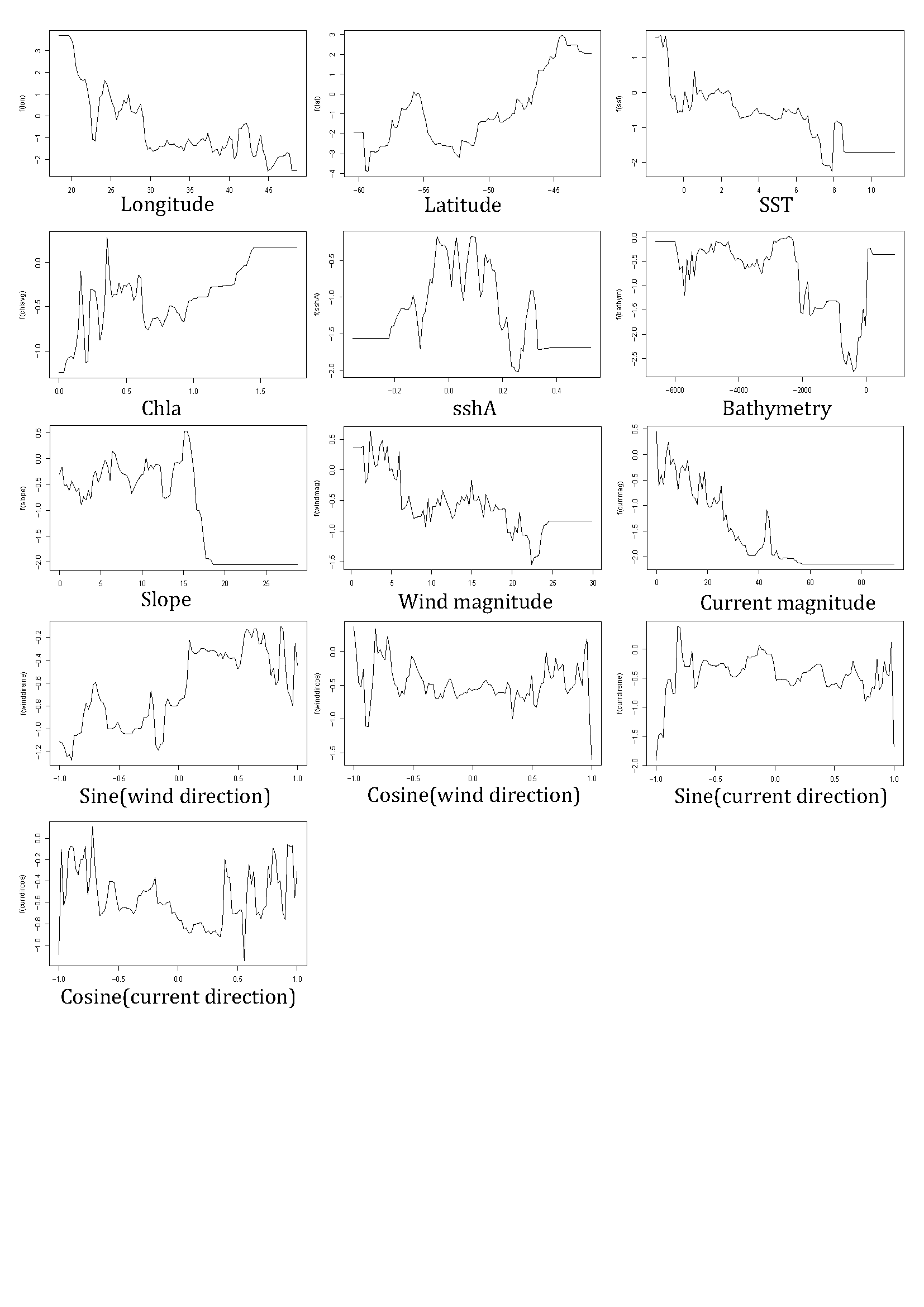


Additional Figure S4: Winter high-density Antarctic fur seal (*Arctocephalus gazella*; HD_AFS) females’ response curves of each of the environmental predictor variables for the final Boosted Regression Tree model.


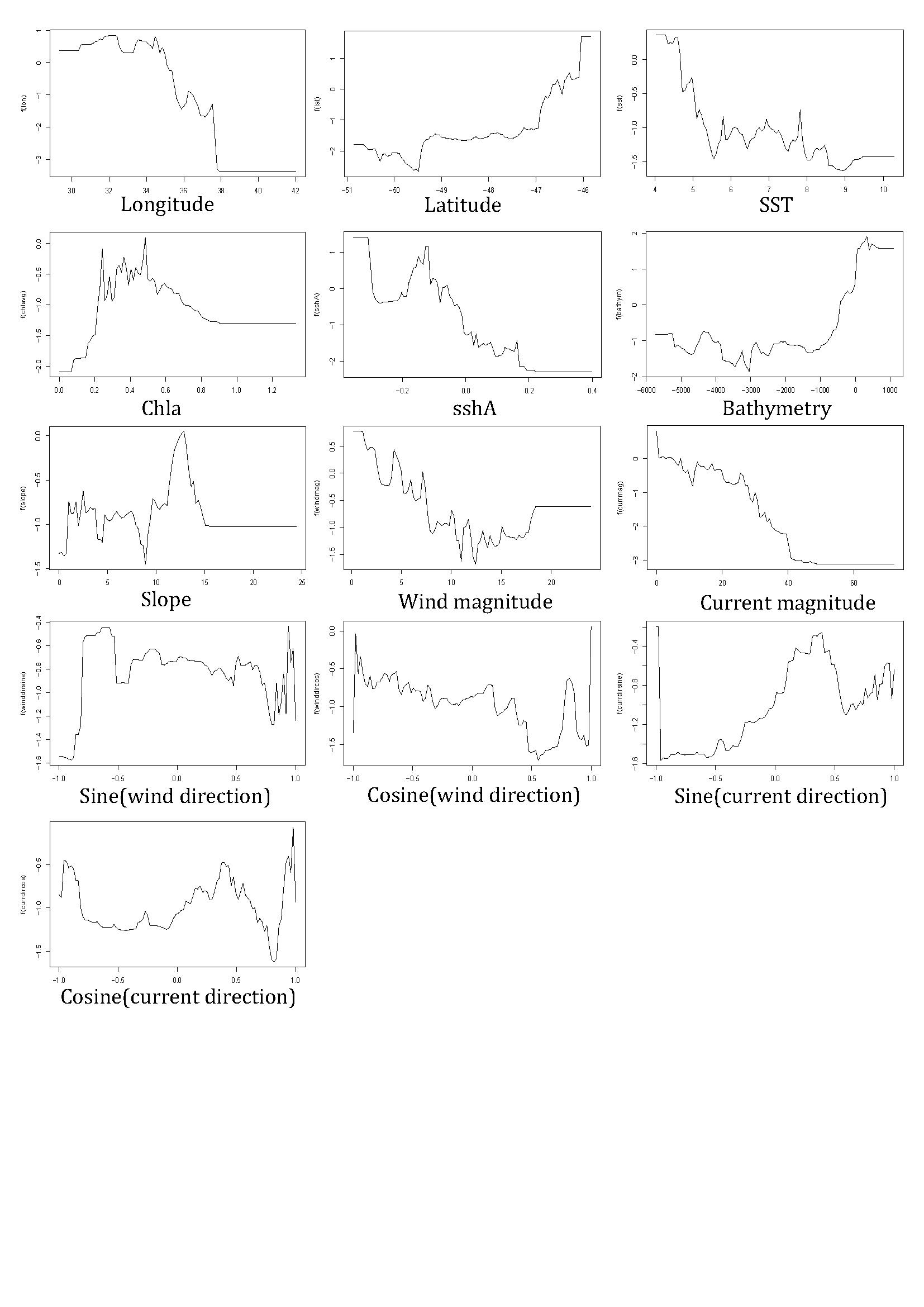


Additional Figure S5: Summer high-density Subantarctic fur seal (*Arctocephalus tropicalis*; HD_SAFS) females’ response curves of each of the environmental predictor variables for the final Boosted Regression Tree model.


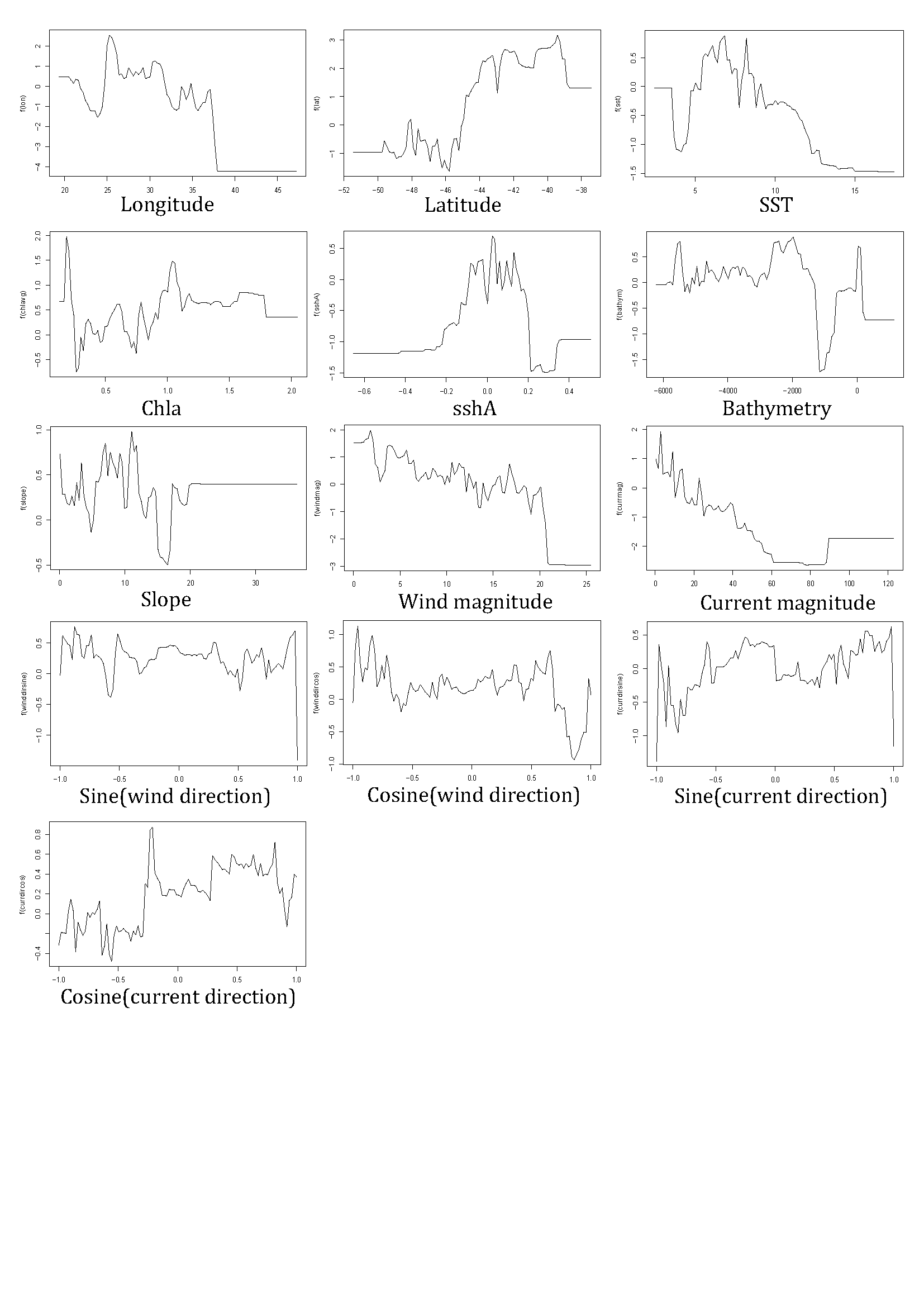


Additional Figure S6: Winter high-density Subantarctic fur seal (*Arctocephalus tropicalis*; HD_SAFS) females’ response curves of each of the environmental predictor variables for the final Boosted Regression Tree model.


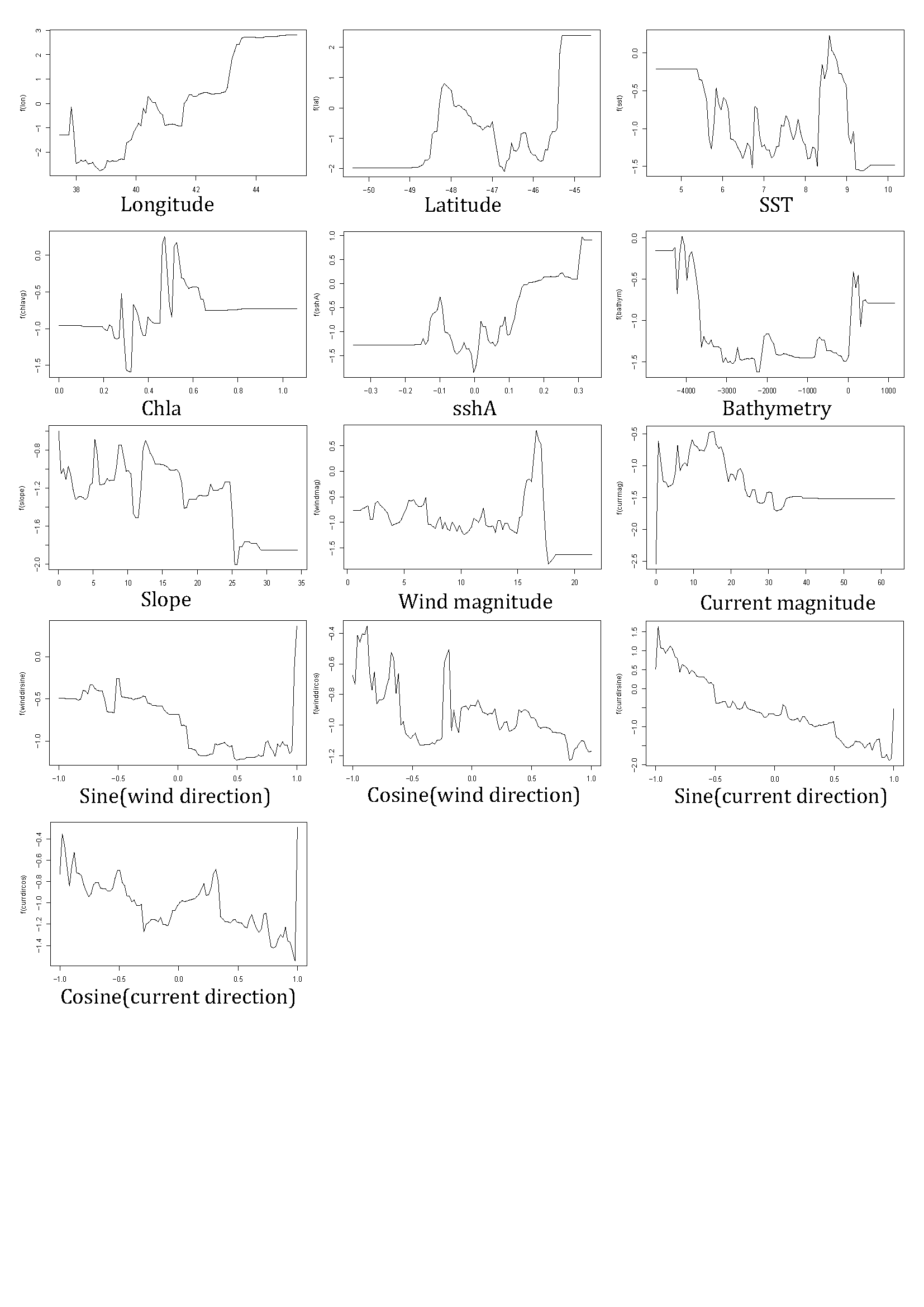


Additional Figure S7: Summer low-density Subantarctic fur seal (*Arctocephalus tropicalis*; LD_SAFS) females’ response curves of each of the environmental predictor variables for the final Boosted Regression Tree model.


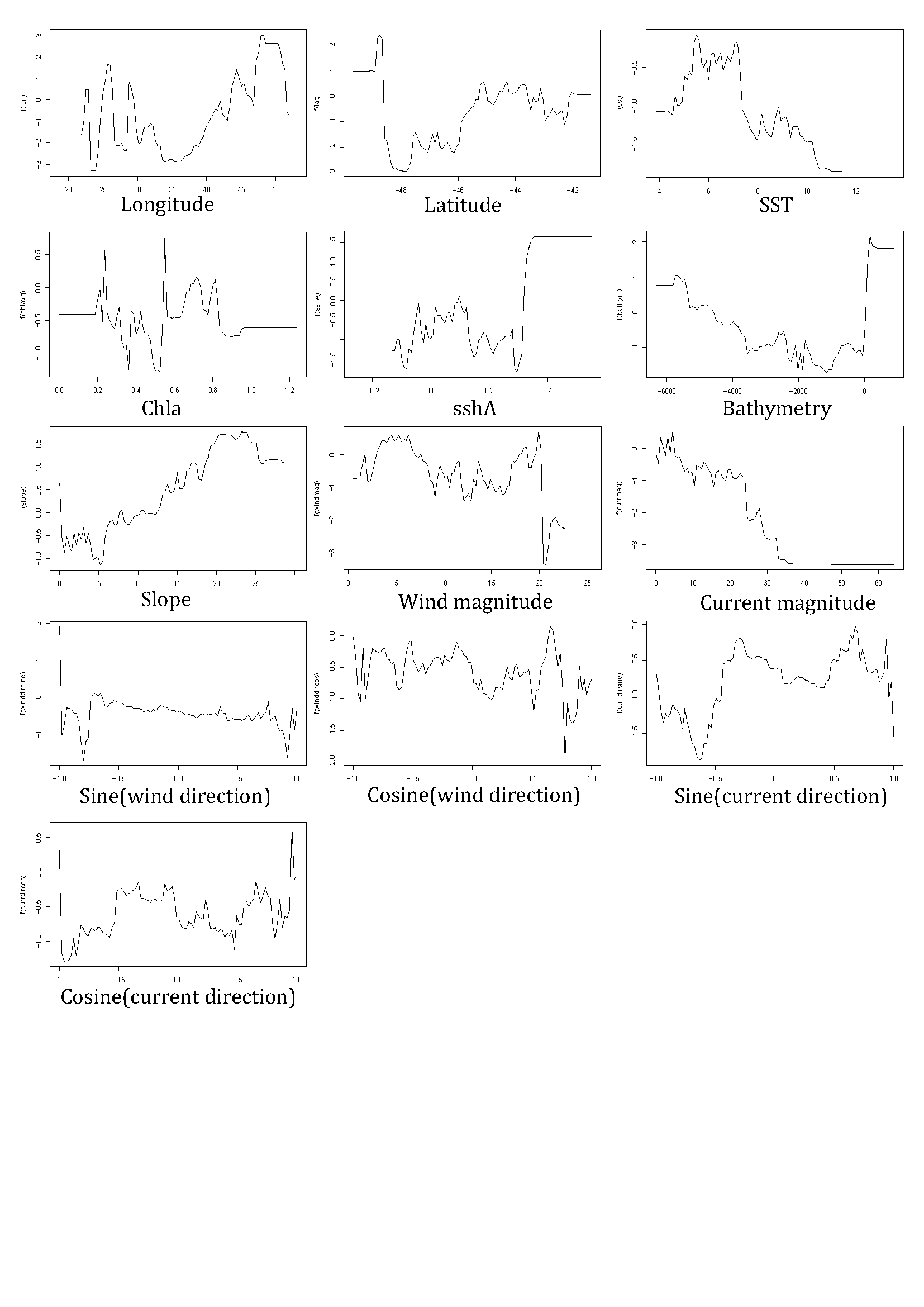


Additional Figure S8: Winter low-density Subantarctic fur seal (*Arctocephalus tropicalis*; LD_SAFS) females’ response curves of each of the environmental predictor variables for the final Boosted Regression Tree model.
